# Editorial: Application of Protective Cultures and Bacteriocins for Food Biopreservation

**DOI:** 10.3389/fmicb.2019.01561

**Published:** 2019-07-04

**Authors:** Riadh Hammami, Ismail Fliss, Aldo Corsetti

**Affiliations:** ^1^School of Nutrition Sciences, Faculty of Health Sciences, University of Ottawa, Ottawa, ON, Canada; ^2^Department of Food Science, Faculty of Agriculture and Food Sciences, Université Laval, Québec, QC, Canada; ^3^Faculty of Bioscience and Technology for Food, Agriculture and Environment, University of Teramo, Teramo, Italy

**Keywords:** protective cultures, bacteriocins, food biopreservation, Lactic acid bacteria, foodborne pathogens, food microbiota

The use of microorganisms and their metabolites for the preservation of foods began in prehistory. Lactic acid bacteria (LAB) are generally recognized as safe for this purpose. They produce an array of antimicrobial substances such as organic acids, diacetyl, acetoin, hydrogen peroxide, reuterin, reutericyclin, and bacteriocins, all of which inhibit foodborne pathogens and spoilage microorganisms. The efficacy of bacteriocins as well as their producing strains for inhibiting several bacterial pathogens has been shown in different food matrices including cheese, meat and vegetables. Nowadays, Protective cultures and bacteriocins are considered as promising alternative. They hold the promise of ensuring the quality and safety of ready-to-eat, extended-shelf-life, fresh-tasting, and minimally processed foods without chemical preservatives. This editorial provides comprehensive overview of bacterial cultures, bacteriocins, and other metabolites that have shown promise for use as antimicrobial bio-preservatives in foods in general. Articles describing novel analytical technologies, strategies to reduce or eliminate pathogens in food systems or emerging technologies for the production or use of protective cultures or their bacteriocins are presented.

The number of bacteriocins and bacteriocin-producing LAB reported to inhibit foodborne pathogens in dairy products including milk, yogurt, and cheeses has grown steadily since the approval of nisin and pediocin for commercial use in foods. In this Research Topic, Silva et al. review the most recent trends in their use in dairy products and discuss critically the efficacy and the pros and cons of their application.

Elimination of foodborne *Listeria monocytogenes* is the focus of *in vitro* studies by Saraoui et al., who are investigating the antagonistic effect of *Lactococcus piscium* CNCM I-4031 against several strains of the pathogen in co-culture or in HT-29 cell culture. Teixeira et al. are studying the impact of pressure and RTE meat microbiota with or without nisin and rosemary oil on the survival of *Listeria* during storage at refrigerator temperatures. Two articles on a similar subject discuss the role of microbial communities as a potential major influence on bacteriocin efficacy and pathogen survival in RTE foods (Ortega Blázquez et al.; Teixeira et al.).

One of the more recent developments included in this Research Topic is the incorporation of bacteriocins into polymer films and coatings applied directly onto food surfaces and packaging materials, also reviewed by Silva et al. The impact of activated plastic and chitosan films loaded with enterocin AS-48 or divergicin M35 on the dynamics of commensal bacteria and the survival of pathogens in sea bream filets and cold smoked salmon is evaluated in two of the articles included (Benabbou et al.; Ortega Blázquez et al.).

The broad-spectrum antimicrobial compound known as reuterin (3-hydroxypropionaldehyde, produced by *Lactobacillus reuteri* from glycerol) is used as a preservative on pre-washed and fresh-cut vegetables (lettuce) in a study by Asare et al., also included in this topic.

Of particular interest are the modes of action of bacteriocins, which differ markedly from those of antibiotics. The inhibitory potential and associated mechanisms of LAB strains in co-culture with enterohemorrhagic *E. coli* (EHEC) in a meat-based medium are presented in a recent article (Orihuel et al.). These authors show also that the activity of the bacteriocin produced by *Enterococcus mundtii* CRL35 is due to an inhibitory mechanism that is more robust. A cell-to-cell contact mechanism is described in studies by Orihuel et al.; Saraoui et al., and novel transcriptomic data relating to a mechanism that regulates hydrogen peroxide production by *Lactococcus garvieae* are presented in an article on inhibition of *Staphylococcus aureus* (Delpech et al.). Most bacteriocins interact specifically with bacterial membranes and kill microbial cells by causing leakage of cellular contents. Wayah and Philip have described pentocin MQ1, a novel bacteriocin produced by a strain of *Lactobacillus pentosus* CS2 and having a bactericidal mode of action mediated by pore formation. The application of pentocin MQ1 to preserving fresh bananas is also presented. Zhang et al. have examined the role of oxidative stress in the efficacy of the yeast *Cryptococcus laurentii* as an inhibitor of the fruit-spoiling fungus *Penicillium expansum*. The authors report an oxidative stress response that appears to activate an antioxidant enzyme system (catalase and superoxide dismutase). Using glutathione to enhance ROS scavenging, they show that *C. laurentii* viability under conditions of oxidative stress can be improved.

The discovery and full characterization of novel bacteriocins with little or no homology to known bacteriocins involves lengthy research. Yi et al. have combined complete genome and peptidome data in an effective approach to discovering novel bacteriocins. They describe the bacteriocin BM1122 from *L. crustorum* MN047 as proof of concept.

An important focus in this Research Topic is the use of emerging technologies for the large-scale production of bacteriocins, a major obstacle to the commercial viability of using these natural preservatives in food or pharma applications. Mesa-Pereira et al. review the different expression systems for bacteriocin production using *E. coli* as host and identify the most important features to guarantee successful production of a range of bacteriocins. Bédard and Biron have summarized synthetic approaches that have been developed for the large-scale production of class IIa bacteriocins and S-linked glycopeptides obtained from LAB. They also highlight the recent advances in peptide engineering and synthesis that have made the chemical synthesis of bacteriocins more cost-competitive.

In summary, the articles included in this Research Topic provide an overview of the current and future trends in food industrial applications of bacteriocins and bacteriocin-producing bacteria.

## Author Contributions

RH wrote the manuscript with support from IF and AC. All authors read and approved the final manuscript.

### Conflict of Interest Statement

The authors declare that the research was conducted in the absence of any commercial or financial relationships that could be construed as a potential conflict of interest.

